# Tighter or less tight glycaemic targets for women with gestational diabetes mellitus for reducing maternal and perinatal morbidity: A stepped-wedge, cluster-randomised trial

**DOI:** 10.1371/journal.pmed.1004087

**Published:** 2022-09-08

**Authors:** Caroline A. Crowther, Deborah Samuel, Ruth Hughes, Thach Tran, Julie Brown, Jane M. Alsweiler, on behalf of the TARGET Study Group

**Affiliations:** 1 Liggins Institute, University of Auckland, Auckland, New Zealand; 2 Department of Obstetrics and Gynaecology, Christchurch Women’s Hospital, University of Otago, Christchurch, New Zealand; 3 Osteoporosis and Bone Biology, Garvan Institute of Medical Research, Sydney, Australia; 4 Department of Paediatrics: Child and Youth Health, University of Auckland, Auckland, New Zealand; University of Manchester, UNITED KINGDOM

## Abstract

**Background:**

Treatment for gestational diabetes mellitus (GDM) aims to reduce maternal hyperglycaemia. The TARGET Trial assessed whether tighter compared with less tight glycaemic control reduced maternal and perinatal morbidity.

**Methods and findings:**

In this stepped-wedge, cluster-randomised trial, identification number ACTRN12615000282583, 10 hospitals in New Zealand were randomised to 1 of 5 implementation dates. The trial was registered before the first participant was enrolled. All hospitals initially used less tight targets (fasting plasma glucose (FPG) <5.5 mmol/L (<99 mg/dL), 1-hour <8.0 mmol/L (<144 mg/dL), 2 hour postprandial <7.0 mmol/L (<126 mg/dL)) and every 4 months, 2 hospitals moved to use tighter targets (FPG ≤5.0 mmol/L (≤90 mg/dL), 1-hour ≤7.4 mmol/L (≤133 mg/dL), 2 hour postprandial ≤6.7 mmol/L) (≤121 mg/dL). Women with GDM, blinded to the targets in use, were eligible. The primary outcome was large for gestational age. Secondary outcomes assessed maternal and infant health. Analyses were by intention to treat. Between May 2015 and November 2017, data were collected from 1,100 women with GDM (1,108 infants); 598 women (602 infants) used the tighter targets and 502 women (506 infants) used the less tight targets. The rate of large for gestational age was similar between the treatment target groups (88/599, 14.7% versus 76/502, 15.1%; adjusted relative risk [adjRR] 0.96, 95% confidence interval [CI] 0.66 to 1.40, *P* = 0.839). The composite serious health outcome for the infant of perinatal death, birth trauma, or shoulder dystocia was apparently reduced in the tighter group when adjusted for gestational age at diagnosis of GDM, BMI, ethnicity, and history of GDM compared with the less tight group (8/599, 1.3% versus 13/505, 2.6%, adjRR 0.23, 95% CI 0.06 to 0.88, *P* = 0.032). No differences were seen for the other infant secondary outcomes apart from a shorter stay in intensive care (*P* = 0.041). Secondary outcomes for the woman showed an apparent increase for the composite serious health outcome that included major haemorrhage, coagulopathy, embolism, and obstetric complications in the tighter group (35/595, 5.9% versus 15/501, 3.0%, adjRR 2.29, 95% CI 1.14 to 4.59, *P* = 0.020). There were no differences between the target groups in the risk for pre-eclampsia, induction of labour, or cesarean birth, but more women using tighter targets required pharmacological treatment (404/595, 67.9% versus 293/501, 58.5%, adjRR 1.20, 95% CI 1.00 to 1.44, *P* = 0.047). The main study limitation is that the treatment targets used may vary to those in use in some countries.

**Conclusions:**

Tighter glycaemic targets in women with GDM compared to less tight targets did not reduce the risk of a large for gestational age infant, but did reduce serious infant morbidity, although serious maternal morbidity was increased. These findings can be used to aid decisions on the glycaemic targets women with GDM should use.

**Trial registration:**

The Australian New Zealand Clinical Trials Registry (ANZCTR). ACTRN12615000282583.

## Abstract 2

Caroline A. Crowther and colleagues investigate whether tighter or less tight glycaemic targets for women with gestational diabetes mellitus reduce maternal and perinatal morbidity in New Zealand.

## Author summary

**Why was this study done?:**

- Gestational diabetes mellitus (GDM) is an increasing global problem with significant short- and long-term health complications for the mother and her infant.
- Treatment for GDM aims to reduce maternal hyperglycaemia although it remains uncertain how tight glycaemic control should be to minimise maternal and perinatal risks.

**What did the researchers do and find?:**

- We performed a stepped-wedge, cluster-randomised trial that included 10 hospitals who were allocated at 4 monthly intervals, in clusters of 2, to change from use of less tight glycaemic targets to use of tighter glycaemic targets in the treatment of women with GDM.
- A total of 1,100 women (1,108 infants) diagnosed with GDM between 22 to 34 weeks’ gestation were enrolled in the trial and the glycaemic targets in use at their hospital were used to control their blood glucose concentrations.
- Use of the tighter glycaemic targets did not reduce the risk of the infant being born large for gestational age but did reduce the risk of serious infant morbidity, although serious maternal morbidity was increased.

**What do these findings mean?:**

- These results provide high-quality evidence for women with GDM and their health practitioners to make informed clinical decisions on the choice of treatment targets to use for glycaemic control.

## Introduction

Gestational diabetes mellitus (GDM), defined by the World Health Organization as “carbohydrate intolerance with onset or recognition during pregnancy” [1], is a significant and increasing health problem globally [2]. Women with GDM are more likely to give birth to a large for gestational age infant, and this risk directly correlates with the severity of the maternal hyperglycaemia [3]. Infants born large for gestational age are at high risk of later development of obesity, diabetes, and the metabolic syndrome [4], so effective treatments that can reduce maternal hyperglycaemia, optimise fetal growth, and reduce the risk of being born large for gestational age have the potential for both short- and long-term health benefits. Dietary and lifestyle advice given to women with GDM and pharmacological therapy when needed that aims to lower maternal fasting and postprandial blood glucose concentrations, improves maternal and perinatal health [5,6], and these are now recommended practices worldwide [7]. The optimal glycaemic targets for women with GDM to use however remain unclear as the targets currently recommended are all higher than the normal blood glucose concentrations from mid pregnancy in women without diabetes [8].

Very few studies have compared the use of different glycaemic targets. In a systematic review of glycaemic targets for women with GDM, no randomised trials comparing different glycaemic targets were identified [9]. From the 26 observational studies included that were all considered of low quality, a fasting plasma glucose (FPG) of <5.0 mmol/L (<90 mg/dL) was associated with benefits of a reduced risk of pre-eclampsia, fewer large for gestational age infants, less neonatal hyperbilirubinaemia, and less neonatal hypoglycaemia [9]. The Cochrane review of different intensities of glycaemic control for women with GDM [10] identified a single, randomised trial reporting data on 171 women that compared the use of glycaemic targets of fasting 5.8 mmol/L (104 mg/dL) and 1-hour postprandial of 7.8 mmol/L (140 mg/dL) with fasting 5.0 mmol/L (90 mg/dL) and 1-hour postprandial of 6.7 mmol/L (121 mg/dL) [11]. This trial did not find any differences between the glycaemic target groups for infant birth weight or for the few maternal outcomes reported but did find that the tighter glycaemic targets were associated with an increased use of pharmacological therapy [11].

Clinical practice guideline recommendations on the glycaemic targets to use for women with GDM vary worldwide, as all rely on consensus due to the paucity of high-quality evidence [12–16]. In New Zealand, the recommended glycaemic targets for women with GDM since 1998 had been a fasting glucose <5.5 mmol/L (<99 mg/dL), 1-hour postprandial <8.0 mmol/L (<144 mg/dL), and-2 hour postprandial <7.0 mmol/L (<126 mg/dL) [17]. With increasing concerns that these targets were not tight enough to minimise the maternal and perinatal risks associated with GDM, in 2014 the updated Ministry of Health Clinical Practice Guidelines “Screening, diagnosis and management of gestational diabetes in New Zealand” recommended, as good practice, to adopt tighter glycaemic targets (fasting ≤5.0 mmol/L (≤90 mg/dL), 1-hour postprandial ≤7.4 mmol/L (≤133 mg/dL), 2-hour postprandial ≤6.7 mmol/L (<121 mg/dL) [12,16]. The conduct of a randomised controlled trial to compare tighter with less tight glycaemic control in women diagnosed with gestational diabetes to assess the impact on maternal and infant health was also strongly recommended [16]. There have been calls worldwide by others too for randomised trials to assess different targets for maternal glycaemic control in women with GDM [9,10,12,18].

The aim of the TARGET Trial was to assess whether tighter targets (FPG ≤5.0 mmol/L (≤90 mg/dL), 1-hour ≤7.4 mmol/L (≤133 mg/dL), 2-hour postprandial ≤6.7 mmol/L (≤121 mg/dL) for glycaemic control in women with GDM compared with less tight targets (FPG <5.5 mmol/L (≤99 mg/dL), 1-hour <8.0 mmol/L (≤144 mg/dL), 2-hour postprandial <7.0 mmol/L (≤126 mg/dL) reduced maternal and perinatal morbidity without adverse health consequences.

## Methods

### Study design and participants

This stepped-wedge, cluster-randomised trial was conducted at 10 maternity hospitals in New Zealand. Human ethics approval was obtained from the Northern A Health and Disability Ethics Committee in New Zealand (14/NTA/163/AMO1) that included a waiver of consent for eligible women being treated for gestational diabetes at the participating hospitals. At the time of planning the trial, hospitals throughout New Zealand were already planning to implement the tighter glycaemic targets recommended in the clinical practice guideline and endorsed by the government. The stepped-wedge cluster design was considered the most appropriate design as it offered the opportunity to evaluate the effects of the implementation of the tighter glycaemic target recommendations across the country, thus facilitating cluster recruitment and creating a logistically feasible design [16,19].

The trial is registered with www.anzctr.org.au, number ACTRN12615000282583. The TARGET Trial study protocol has been published (S1 File) [20]. Women between 22 and 34 weeks’ gestation with GDM diagnosed by a 75 g oral glucose tolerance test (OGTT) in mid-pregnancy (FPG ≥5.5 mmol/L (≥99 mg/dL) or 2-hour ≥9.0 mmol/L (≥162 mg/dL)) [16,17], receiving care at one of the participating hospitals were eligible for inclusion. Women with a known major fetal malformation were not eligible. Eligible women were enrolled into the study by the research coordinator at each hospital. This study is reported as per the CONSORT extension for Cluster Trials guideline (S1 Table) [21].

### Randomisation and masking

Hospitals were paired based on the expected number of women presenting with GDM to make the clusters similar in size and were randomised, in clusters of 2, at 4 monthly intervals to 1 of 5 implementation dates to change from use of the less tight glycaemic targets (FPG <5.5 mmol/L (<99 mg/dL), 1-hour <8.0 mmol/L (<144 mg/dL), 2-hour postprandial <7.0 mmol/L (<126 mg/dL)) to use of the tighter glycaemic targets (FPG ≤5.0 mmol/L (≤90 mg/dL), 1-hour ≤7.4 mmol/L (≤133 md/dL), 2-hour postprandial ≤6.7 mmol/L (≤121 mg/dL)). The dates were concealed from personnel at the hospitals until 2 weeks prior to the target change when training for the implementation of the tight glycaemic targets began at their site. The allocation sequence of the hospitals to the implementation of the tight glycaemic targets was prepared by a statistician using a computer-generated random number table. Enrolled women were advised of the glycaemic targets in use for their hospital.

### Procedures

All hospitals started the trial using the less tight targets. Before the study commenced, the lead investigator and study coordinator met with the clinical leaders in obstetrics, diabetes, and midwifery at each hospital to plan the setup and running of the project and to provide the TARGET Trial Implementation Action Pack. The pack included the trial protocol, a presentation for use in local educational meetings about the study, stickers of the less tight targets to be used in the woman’s blood glucose monitoring booklets, lanyard study cards for relevant hospital staff that gave the treatment targets in use, and posters to display in clinical areas about the less tight targets for glycaemic control in use at the hospital. When the study commenced at the hospital, all health professionals caring for women with GDM were sent a reminder of the less tight targets to use for glycaemic control in women with GDM.

Two weeks prior to a hospital being randomised to implement the tighter glycaemic targets, the lead investigator and study coordinator met again with the hospital staff to plan their change to use of the tighter targets. Updated materials were provided for the site’s TARGET Trial Implementation Action Pack. At the implementation date, all health professionals caring for women with GDM at the hospital were sent a reminder of the tighter targets to use for glycaemic control in women with GDM.

### For both glycaemic target periods

Women with GDM attending the participating hospitals were cared for by their lead maternity carer and the local Diabetes Pregnancy Service according to standard practice at each hospital. Glycaemic targets recommended were those the hospital was using when the women first attended for diabetes care and remained unchanged until their birth. Standard practice included appropriate dietary and lifestyle advice, blood glucose monitoring, and pharmacological treatment as needed [16]. Care of the infant after birth was according to the hospital’s protocol including for blood glucose monitoring.

### Data collection

At each of the participating sites, the research coordinator collected the pregnancy, birth, and postnatal study outcome data from the case records of the women enrolled and their infants, up to the time of primary hospital discharge after the birth. Data were transferred to the data management centre at the Liggins Institute, University of Auckland and stored in a password-protected database.

### Outcomes

The primary outcome measure was the incidence of large for gestational age, defined as birth weight >90th centile using growth charts adjusted for gestational age and infant sex [22].

Prespecified secondary outcomes for the infant prior to hospital discharge were a composite of serious health outcomes, defined as perinatal death or birth trauma (nerve palsy, bone fracture), or shoulder dystocia; gestational age at birth; other measures of body size at birth (weight, length and head circumference and z scores, macrosomia defined as birth weight ≥4 kg, small for gestational age defined as a birth weight <10th centile); use of respiratory support; hypoglycaemia; hyperbilirubinaemia; neonatal intensive care unit admission and length of stay; and length of postnatal stay.

Prespecified secondary outcomes for the women were a composite of serious health outcomes [23], (defined as one or more of maternal death, pulmonary oedema, eclampsia, stroke, adult respiratory distress syndrome, cardiac arrest, respiratory arrest, placental abruption, haemolysis, coagulopathy, major postpartum haemorrhage, deep vein thrombosis, or pulmonary embolus requiring anticoagulant therapy), pre-eclampsia, induction of labour, cesarean section, use of pharmacological treatment for GDM, hypoglycaemia, need for antenatal admission and length of stay, length of postnatal stay, and breast feeding at hospital discharge.

### Statistical analysis

Our pre-planned sample size of 1,080 participants from 10 hospitals over 6 periods of 4 months would provide 90% power at 5% level of significance (2-sided) to detect a treatment difference of 6% in the proportion of large for gestational age babies, from 13% [5] using the less tight glycaemic treatment targets to 7% [6] using the tighter glycaemic treatment targets, assuming an intra-cluster correlation coefficient of 0.05 [24]. Statistical analyses followed a prespecified analysis plan by an independent statistician using an intention-to-treat approach with SAS software version 9.4 (SAS Institute, Cary, North Carolina, United States of America). Outcome data in the tighter glycaemic target period were compared with outcome data in the less tight glycaemic target period.

Generalized linear mixed-effects models were used to determine the main treatment effect, with a random effect for hospital groups, and fixed effects for the intervention and the time interval, in months, between the date the assigned treatment targets were initiated and the gestational age the woman was diagnosed with GDM. Time was included in the model to account for secular trends over time as the study design induces an association between time and outcome of interest [24]. Binary outcomes were analysed using a log Poisson mixed-effects model with robust variance estimation to obtain relative risk (RR) and its corresponding 95% confidence interval (CI). If the number of subjects experiencing the outcome was considered too small for the planned analysis, a mid-P exact test was performed. Count outcomes were analysed using a negative binomial model if data overdispersion was evident and reported ratio of means (95% CI). Continuous outcomes were analysed using a linear mixed-effects model and the effect size was reported as mean difference (95% CI). Analyses for infant outcomes accounted for the clustering effect of infants within mothers.

As adjustment for potential confounding covariates has been recommended to improve the efficacy of the analysis, providing stronger and more precise evidence of a treatment effect [25], the adjusted analyses made adjustment for the prespecified covariate of gestational age at time of the OGTT. We calculated the adjusted number needed to treat to benefit (NNTB) and 95% CI for binary outcomes with adjustment for covariates in randomised controlled trials using the SAS macro *%nnt* [26]. Prespecified exploratory analyses were undertaken to further adjust for covariates that showed evidence of important imbalance between the study groups and were related to the outcome of interest. These additional covariates were BMI, ethnicity, and history of GDM. A post hoc sensitivity analysis was conducted, as requested by the statistical reviewer, that accounted for 2, additional, random effects; the time-by-hospital interaction and the intervention-by-hospital interaction. If the model with multiple random effects failed to converge due to lack of variability, a simpler model with only random effect for the hospital groups was used [27]. A 2-sided *p*-value of less than 0.05 was considered statistically significant. The study was overseen by a data safety monitoring committee. No interim analyses were planned or undertaken.

## Results

**Fig 1:**
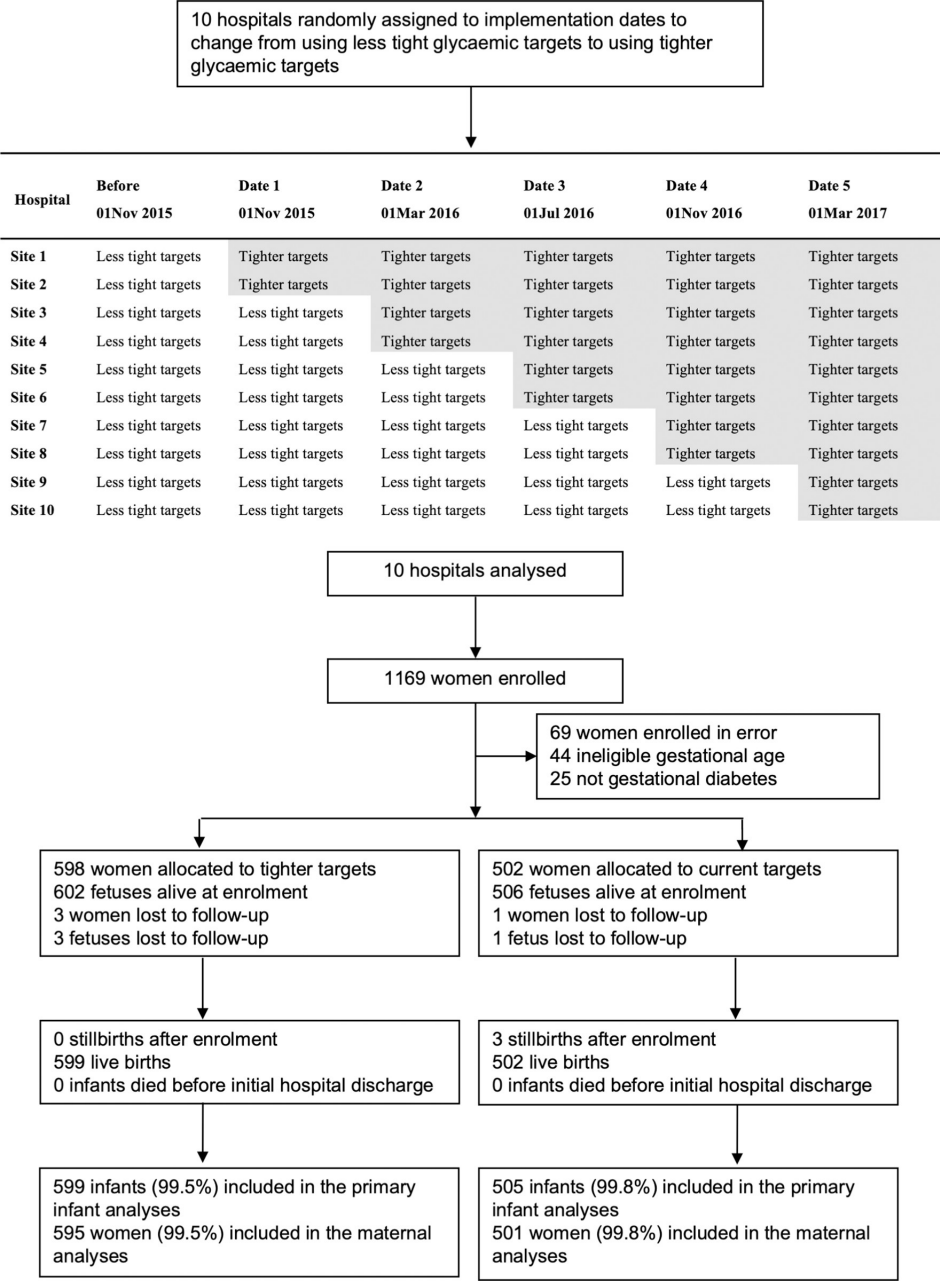
Trial profile. The shaded area indicates the change of a site to the tighter targets and the time of that change.

Between May 29, 2015 and November 7, 2017, 1,100 women (1,108 infants) were enrolled at the 10 hospitals participating in the trial. Of these 1,080 women, 598 (55%) women (602 infants) were included while their hospital was allocated to the use of tighter targets and 502 (45%) women (506 infants) while their hospital was allocated to the use of less tight targets (Fig 1).

Maternal characteristics at baseline were similar between the 2 groups for gestational age at enrolment and oral glucose tolerance test results (Table 1). Fewer women in the tighter target group were likely to self-identify as European and to have a previous history of gestational diabetes but their BMI was higher than women in the less tight target group (Table 1). Three women (<1%) in the tighter target group and 1 woman (<1%) in the less tight group were lost to follow up, so 595 women (599 infants) in the tighter target group and 501 women (505 infants) in the less tight group were included in the analyses (Fig 1).

**Table 1:** Baseline maternal characteristics at trial entry.

Characteristics	Tighter targets *n* = 598 (n/N [%])	Less tight targets *n* = 502 (n/N [%])
Primiparous	231 (38.6)	205 (40.8)
BMI (kg/m^2^)†	31.7 (27.6–37.9)	30.9 (27.6–36.7)
**BMI category:**		
Normal	62 (10.5)	59 (12.1)
Overweight	161 (27.3)	151 (31.1)
Obese	366 (62.1)	276 (56.8)
**Ethnicity:** ‡		
European	201 (33.6)	253 (50.4)
Pacifica	81 (13.5)	42 (8.4)
Māori	92 (15.4)	56 (11.2)
Asian	210 (35.1)	143 (28.5)
Other	14 (2.3)	8 (1.6)
Gestational age at entry (weeks, days)^§^	28.4 ± 2.5	28.2 ± 2.5
Smoking status	63 (10.5)	50 (10.0)
Previous perinatal death	18 (4.9)	11 (3.7)
Previous gestational diabetes	90 (24.5)	94 (31.6)
Oral glucose tolerance test; fasting result (mmol/L)†	5.2 (4.6–5.7)	5.0 (4.5–5.7)
Oral glucose tolerance test 2-hour result (mmol/L)†	9.4 (9.0–10.2)	9.5 (9.0–10.2)

Values are number (%), unless otherwise indicated.

^†^Values are Medians (IQR).

^**‡**^Ethnicity as reported by the participant and prioritised according to the New Zealand Ministry of Health classification. (Ministry of Health. Ethnicity New Zealand Standard Classification 2005 V2.1.0. Statistics New Zealand; website: http://aria.stats.govt.nz/aria/).

^§^Plus-minus values are means ± SD.

BMI, body mass index.

The primary outcome of large for gestational age occurred in 88 of 599 (14.7%) infants in the tighter target group and in 76 of 502 (15.1%) infants in the less tight target group (adjusted relative risk (adjRR) 0.96, 95% CI 0.66 to 1.40, *P* = 0.839) (Table 2). Prespecified analyses adjusting additionally for maternal BMI, ethnicity, and previous history of GDM found the incidence of a large for gestational age infant was similar to the primary analyses (adjRR 0.99, 95% CI 0.67 to 1.46, *P* = 0.968). For the secondary outcome measures for infant body size of birth weight, birth weight z scores, the incidence of macrosomia, or the risk of being small for gestational age, there were no differences between the 2 target groups (Table 2).

**Table 2:** Primary outcome and secondary birth size outcomes for the infant.

Outcomes	Tighter targets (*n* = 599)	Less tight targets (*n* = 502)	Treatment effects+ (95% CI)	*p*-value	Adjusted+* treatment effects (95% CI)	Adjusted* *p*-value	Adjusted +† treatment effects (95% CI)	Adjusted+† *p*-value
Primary outcome								
Large for gestational age	88/599 (14.7%)	76/502 (15.1%)	0.97 (0.66–1.41)	0.858	0.96 (0.66–1.40)	0.839	0.99 (0.67–1.46)	0.968
Secondary birth size outcomes								
Birth weight (g)^1^	3367.95 (537.94)	3359.29 (557.68)	21.50 (−58.19–101.19)	0.597	20.04 (−58.64–98.73)	0.618	19.43 (−56.03–94.88)	0.614
Birth weight (z score)^1^	0.32 (1.02)	0.22 (1.02)	0.11 (−0.04–0.26)	0.160	0.11 (−0.04–0.26)	0.156	0.11 (−0.03–0.24)	0.127
Small for gestational age	29/599 (4.8%)	31/502 (6.2%)	0.63 (0.33–1.20)	0.162	0.64 (0.33–1.21)	0.169	0.61 (0.32–1.18)	0.142
Macrosomia#	61/599 (10.2%)	54/502 (10.8%)	1.11 (0.72–1.73)	0.632	1.10 (0.71–1.72)	0.662	1.08 (0.68–1.71)	0.754
Length at birth—large for gestational age	120/556 (21.6%)	100/438 (22.8%)	0.98 (0.71–1.36)	0.907	0.98 (0.71–1.36)	0.916	1.00 (0.72–1.38)	0.983
Length at birth (cm)^1^	50.81 (2.65)	50.91 (2.65)	0.13 (−0.28–0.53)	0.532	0.12 (−0.29–0.52)	0.573	0.11 (−0.29–0.51)	0.584
Birth length (z score)^1^	0.57 (1.01)	0.56 (1.02)	0.12 (−0.04–0.28)	0.132	0.12 (−0.04–0.27)	0.135	0.12 (−0.03–0.27)	0.114
Head circumference at birth—large for gestational age	112/560 (20.0%)	84/456 (18.4%)	1.00 (0.71–1.42)	0.982	1.00 (0.71–1.42)	0.997	1.00 (0.70–1.42)	0.985
Birth head circumference (cm)^1^	34.58 (1.57)	34.50 (1.72)	0.03 (−0.22–0.28)	0.823	0.02 (−0.23–0.26)	0.885	0.01 (−0.23–0.25)	0.924
Birth head circumference (z score)^1^	0.40 (1.05)	0.28 (1.10)	0.07 (−0.09–0.24)	0.371	0.07 (−0.09–0.23)	0.386	0.07 (−0.08–0.22)	0.374

Data presented as number (%), and the treatment effects are relative risk (95% CI) estimated from the generalised linear mixed-effects model, with a random effect for hospital groups, and fixed effects for the intervention and time interval (months) between the assigned targets initiated and a woman recruited, unless otherwise indicated.

^**+**^Adjusted for clustering effect of infants within mothers.

*Adjusted for gestational age at oral glucose tolerance test (weeks).

†Adjusted for gestational age at oral glucose tolerance test, ethnicity, BMI, and history of gestational diabetes.

^1^Data presented as mean (SD), and the treatment effects are mean difference (95% CI).

^#^Defined as a birth weight ≥4 kg.

CI, confidence interval.

The composite of a serious health outcome for the infant, defined as perinatal death or birth trauma (nerve palsy, bone fracture) or shoulder dystocia, occurred in 8 of 599 (1.3%) infants in the tight target group and in 13 of 505 (2.6%) infants in the less tight target group (adjRR 0.33, 95% CI 0.10 to 1.08, *P* = 0.068) (Table 3). With additional adjustment for maternal BMI, ethnicity, and previous history of GDM, the risk of a serious health outcome for the infant was apparently reduced (adjRR 0.23, 95% CI 0.06 to 0.88, *P* = 0.032; NNTB 47, 95% CI 25 to 430). There were no (0%) stillbirths in the tighter target group compared with 3 (0.59%) in the less tight target group (*P* = 0.095). There were no neonatal deaths in either target group. The tighter target group had 8 of 599 (1.3%) cases of shoulder dystocia compared with 10 of 502 (2.0%) in the less tight group (*P* = 0.198). There were no reports of birth trauma in either target group.

**Table 3:** Other secondary infant outcomes.

Outcomes	Tighter targets (*n* = 599)	Current targets (*n* = 505)	Treatment effects+ (95% CI)	*p*-value	Adjusted+* Treatment effects (95% CI)	Adjusted* *p*-value	Adjusted +† treatment effects (95% CI)	Adjusted+† *p*-value
Gestational age at birth (weeks)^1^	38.44 (1.30)	38.61 (1.42)	−0.12 (−0.32–0.07)	0.219	−0.13 (−0.32–0.07)	0.200	−0.12 (−0.32–0.07)	0.225
Composite of serious health outcome	8/599 (1.3%)	13/505 (2.6%)	0.34 (0.10–1.10)	0.072	0.33 (0.10–1.08)	0.068	0.23 (0.06–0.88)	0.032
Stillbirth	0/599 (0.0%)	3/505 (0.6%)	N/A	0.095^	N/A	N/A	N/A	N/A
Neonatal death	0/599 (0.0%)	0/502 (0.0%)	N/A	N/A	N/A	N/A	N/A	N/A
Birth trauma	0/599 (0.0%)	0/502 (0.0%)	N/A	N/A	N/A	N/A	N/A	N/A
Shoulder dystocia	8/599 (1.3%)	10/502 (2.0%)	0.45 (0.13–1.55)	0.208	0.44 (0.13–1.52)	0.198	0.27 (0.07–1.07)	0.062
Use of respiratory support	43/599 (7.2%)	29/502 (5.8%)	1.11 (0.62–1.98)	0.716	1.13 (0.63–2.01)	0.684	1.16 (0.65–2.07)	0.622
Hypoglycaemia	169/599 (28.2%)	127/502 (25.3%)	0.93 (0.69–1.23)	0.594	0.92 (0.69–1.23)	0.593	0.94 (0.70–1.25)	0.656
Hyperbilirubinemia	27/599 (4.5%)	25/502 (5.0%)	0.84 (0.43–1.65)	0.607	0.83 (0.42–1.64)	0.593	0.78 (0.39–1.56)	0.482
Admission to neonatal intensive care unit	22/599 (3.7%)	20/502 (4.0%)	0.52 (0.25–1.07)	0.076	0.51 (0.25–1.06)	0.073	0.50 (0.23–1.05)	0.068
Length of stay of infants admitted to neonatal intensive care unit^2^	3.49 (4.55)	5.10 (5.72)	0.46 (0.23–0.93)	0.037	0.49 (0.26–0.95)	0.041	0.66 (0.30–1.46)	0.314
Length of Postnatal stay (days)	4.11 (4.75)	4.18 (6.68)	0.95 (0.84–1.08)	0.407	0.95 (0.84–1.08)	0.468	0.95 (0.84–1.08)	0.472

Data presented as number (%), and the treatment effects are relative risk (95% CI) estimated from the generalised linear mixed-effects model, with a random effect for hospital groups, and fixed effects for the intervention and time interval between the assigned targets initiated and a woman recruited, unless otherwise indicated.

^**+**^Adjusted for clustering effect of infants within mothers.

*Adjusted for gestational age at oral glucose tolerance test (weeks).

†Adjusted for gestational age at oral glucose tolerance test, ethnicity, BMI, and history of gestational diabetes.

^1^Data presented as mean (SD), and the treatment effects are mean difference (95% CI).

^2^Data presented as mean (SD), and the treatment effects are mean ratio (95% CI).

^^^Mid-P exact test.

N/A: denotes none or too few events for the analysis to be done.

Gestational age at birth was similar between the glycaemic target groups (Table 3). Admission to the neonatal intensive care unit occurred in 22/599 (3.7%) infants in the tight target group and in 20/502 (4.0%) infants in the less tight target group, (adjRR 0.51, 95% CI 0.25 to 1.06, *P* = 0.073). Length of stay was shorter for infants in the tighter target group (mean days 3.49, SD 4.55 versus 5.10, SD 5.72; adjusted mean ratio 0.49, 95% CI 0.26, 0.95, *P* = 0.041), although with additional adjustment for maternal BMI, ethnicity, and previous history of GDM this was no longer apparent (adjusted mean ratio 0.66, 95% CI 0.30, 1.46, *P* = 0.314) (Table 3). There were no differences between glycaemic target groups for any of the other infant outcomes, use of respiratory support, hypoglycaemia, hyperbilirubinaemia, or length of postnatal stay (Table 3). The post hoc sensitivity analysis yielded similar results for the primary endpoint of large-for-gestational age infants and key secondary outcomes as the primary analysis (S2 Table).

The composite of serious health outcome for the women occurred in 35 of 595 (5.9%) women in the tight target group and in 15 of 501 (3.0%) women in the less tight target group (adjRR 2.29, 95% CI 1.14 to 4.59, *P* = 0.020; number needed to treat to harm 27, 95% CI 15 to 144) (Table 4). After additional adjustment for maternal BMI, ethnicity, and previous history of GDM, the risk of serious health outcome for the women remained significant and similar to the primary analyses (adjRR 2.28, 95% CI 1.11 to 4.67, *P* = 0.025). For the individual components of the composite serious health outcome for the women, there were no differences between the glycaemic target groups, major postpartum haemorrhage accounted for the majority of events in both target groups (26/595, 4.4% in the tighter group versus 12/501, 2.4% in the less tight group) (Table 4).

**Table 4:** Secondary outcomes for the women.

Outcomes	Tighter targets (*n* = 595)	Less tight targets (*n* = 501)	Treatment effects (95% CI)	*p*-value	Adjusted* treatment effects (95% CI)	Adjusted* *p*-value	Adjusted † treatment effects (95% CI)	Adjusted† *p*-value
Serious maternal health outcome	35/595 (5.9%)	15/501 (3.0%)	2.30 (1.15–4.60)	0.019	2.29 (1.14–4.59)	0.020	2.28 (1.11–4.67)	0.025
Maternal death	0/595 (0.0%)	0/501 (0.0%)	N/A	N/A	N/A	N/A	N/A	N/A
Acute pulmonary oedema	0/595 (0.0%)	1/501 (0.2%)	N/A	0.457^^^	N/A	N/A	N/A	N/A
Eclampsia	1/595 (0.2%)	0/501 (0.0%)	N/A	0.543^^^	N/A	N/A	N/A	N/A
Stroke	0/595 (0.0%)	0/501 (0.0%)	N/A	N/A	N/A	N/A	N/A	N/A
Respiratory distress syndrome	0/595 (0.0%)	0/501 (0.0%)	N/A	N/A	N/A	N/A	N/A	N/A
Cardiac arrest	0/595 (0.0%)	1/501 (0.2%)	N/A	0.457^^^	N/A	N/A	N/A	N/A
Respiratory arrest	0/595 (0.0%)	1/501 (0.2%)	N/A	0.457^^^	N/A	N/A	N/A	N/A
Placental abruption	0/595 (0.0%)	0/501 (0.0%)	N/A	N/A	N/A	N/A	N/A	N/A
Haemolysis	2/595 (0.3%)	0/501 (0.0%)	N/A	0.295^^^	N/A	N/A	N/A	N/A
Coagulopathy	6/595 (1.0%)	3/501 (0.6%)	N/A	0.483^^^	N/A	N/A	N/A	N/A
Major postpartum haemorrhage	26/595 (4.4%)	12/501 (2.4%)	1.79 (0.80–4.04)	0.159	1.79 (0.79–4.03)	0.1620	1.76 (0.77–4.05)	0.184
Deep vein thrombosis or pulmonary embolus requiring anticoagulant therapy	2/595 (0.3%)	0/501 (0.0%)	N/A	0.295^^^	N/A	N/A	N/A	N/A
Pre-eclampsia	31/595 (5.2%)	18/501 (3.6%)	1.56 (0.78–3.12)	0.212	1.57 (0.78–3.16)	0.205	1.36 (0.68–2.73)	0.387
Induction of labour	312/595 (52.4%)	259/501 (51.7%)	0.96 (0.78–1.17)	0.681	0.96 (0.78–1.17)	0.681	0.97 (0.79–1.19)	0.759
Cesarean delivery	226/595 (38.0%)	174/501 (34.7%)	1.01 (0.79–1.29)	0.914	1.01 (0.80–1.29)	0.911	0.99 (0.77–1.26)	0.917
Use of pharmacological treatment	404/595 (67.9%)	293/501 (58.5%)	1.22 (1.01–1.47)	0.035	1.20 (1.00–1.44)	0.047	1.18 (0.98–1.41)	0.084
Metformin	330/595 (55.5%)	213/501 (42.5%)	1.25 (1.01–1.54)	0.036	1.25 (1.02–1.54)	0.036	1.19 (0.97–1.48)	0.098
Insulin	204/595 (34.3%)	162/501 (32.3%)	1.46 (1.14–1.86)	0.003	1.46 (1.14–1.86)	0.003	1.49 (1.16–1.91)	0.002
Metformin and insulin	130/598 (21.7%)	82/502 (16.3%)	1.65 (1.20–2.26)	0.002	1.64 (1.19–2.26)	0.002	1.61 (1.16–2.23)	0.005
Maternal hypoglycaemia	5/595 (0.8%)	6/501 (1.2%)	1.24 (0.31–5.01)	0.761	1.23 (0.31–4.93)	0.768	1.53 (0.37–6.32)	0.559
Need for antenatal hospitalisation	117/595 (19.7%)	96/501 (19.2%)	1.09 (0.79–1.51)	0.605	1.09 (0.79–1.52)	0.601	1.05 (0.75–1.46)	0.784
Length of antenatal admission (days)^1^	3.85 (4.06)	3.80 (3.14)	0.95 (0.73–1.23)	0.685	0.96 (0.74–1.25)	0.757	0.93 (0.73–1.20)	0.562
Length of postnatal stay (days)^1^	2.72 (1.85)	2.66 (1.78)	0.97 (0.88–1.07)	0.601	0.98 (0.89–1.07)	0.609	0.98 (0.88–1.08)	0.616
Breastfeeding at discharge	561/595 (94.3%)	479/498 (96.2%)	0.99 (0.85–1.15)	0.882	0.99 (0.85–1.15)	0.881	0.99 (0.85–1.15)	0.884

Data presented as number (%), and the treatment effects are relative risk (95% CI) estimated from the generalised linear mixed-effects model, with a random effect for hospital groups, and fixed effects for the intervention and time interval between the assigned targets initiated and a woman recruited, unless otherwise indicated.

*Adjusted for gestational age at oral glucose tolerance test (weeks).

†Adjusted for gestational age at oral glucose tolerance test, ethnicity, BMI, and history of gestational diabetes.

^1^Data presented as mean (SD), and the treatment effects are mean ratio (95% CI).

^^^Mid-P exact test.

N/A: denotes none or too few events for the analysis to be done.

There were no differences between the glycaemic target groups for the other secondary maternal outcomes of pre-eclampsia (31/595, 5.2% in the tighter targets group versus 18/501, 3.6% in the less tight targets group), induction of labour (312/595, 52.4% in the tighter targets group versus 259/501, 51.7% in the less tight targets group), cesarean birth (226/595, 38.0% in the tighter targets group versus 174/501, 34.7% in the less tight targets group) or breast feeding at hospital discharge (561/595, 94.3% in the tighter targets group versus 479/501, 96.2% in the less tight targets group) (Table 4).

Women in the tighter target group compared to women in the less tight target group were more likely to require pharmacological treatment for glycaemic control (404/595, 67.9% versus 293/501, 58.5%, adjRR 1.20, 95% CI 1.00 to 1.44, *P* = 0.047) (Table 4). This included both the use of metformin (330/595, 55.5% versus 213/501, 42.5%, adjRR 1.25, 95% CI 1.02 to 1.54, *P* = 0.036) and the use of insulin (204/595, 34.3% versus 162/501, 32.3%, adjRR 1.46, 95% CI 1.14 to 1.86, *P* = 0.003) (Table 4). The incidence of maternal hypoglycaemia was low and did not differ between glycaemic target groups, (5/595, 0.8% in the tighter targets group versus 6/501, 1.2% in the less tight targets group) (Table 4).

Need for antenatal admission was similar between the glycaemic target groups (117/595, 19.7% in the tighter targets group versus 96/501, 19.2% in the less tight targets group) as was length of any antenatal stay (mean days 3.85, SD 4.06 versus 3.80, SD 3.14, adjusted mean ratio 0.96, 95% CI 0.74, 1.25, *P* = 0.757) and maternal postnatal length of stay (mean days 2.72, SD 1.85 versus 2.66, SD 1.78, adjusted mean ratio 0.98, 95% CI 0.89, 1.07, *P* = 0.609) (Table 4). The post hoc sensitivity analysis for the maternal outcomes was consistent with those in the primary analysis (S3 Table).

## Discussion

In this stepped-wedge, cluster-randomised trial, conducted at 10 maternity hospitals in New Zealand between May 2015 and November 2017, comparing tighter targets with less tight targets for glycaemic control in women with gestational diabetes, we found no significant difference in the primary outcome of the infants being born large for gestational age between the treatment target groups. Use of tighter glycaemic targets reduced the risk of a serious health outcome for the infant that included death, birth trauma, and shoulder dystocia, and their length of stay in the neonatal intensive care unit was shorter. To be balanced against these benefits was an increased risk of a serious health outcome for the woman when using the tighter targets that included major haemorrhage, coagulopathy, embolism, and obstetric complications, and an increased use of pharmacological therapy to treat maternal hyperglycaemia.

Treatment of GDM to control maternal hyperglycaemia compared to no treatment reduces the risk of a large for gestational age infant and improves other key maternal and perinatal health outcomes [5,6]. Being born large for gestational age is associated with birth trauma, including nerve palsy and bone fracture, but also long-term risks including obesity and diabetes [4]. Women with GDM have a higher risk of a large for gestational age infant, strongly correlated to the degree of maternal hyperglycaemia [3]. We hypothesised that tighter maternal glycaemic control compared to less tight would reduce the risk of a large for gestational age infant, and hence reduce perinatal morbidity and therefore potentially have long-term benefits. However, we found no difference between the use of tighter glycaemic targets and less tight targets on the incidence of large for gestational age or on any other measures of body size.

Previous relevant studies of different glycaemic targets comparing maternal and infant outcomes have been limited [9–11]. A systematic review of 26 non-randomised cohort studies reported that the risk of being born large for gestational age was significantly reduced not only when the fasting glucose was below 5.0 mmol/L (90 mg/dL) and/or the 2-hour postprandial glucose concentrations were ≤6.7 mmol/L (≤121 mg/dL) but also when the fasting glucose was below 5.5 mmol/L (99 mg/dL) and/or the 2-hour postprandial glucose concentrations <7 mmol/L (<126 mg/dL) [9]. These concentrations correspond to the glycaemic cutoffs for both the tighter and less tight targets used in this trial. This suggests that even the use of the less tight targets as used in our trial may provide a maximal beneficial effect on fetal growth and therefore infant body size measures. Our results provide reassurance that the known beneficial effect of a reduced risk of being born large for gestational age when GDM is treated [5,6] can be achieved using our less tight glycaemic targets, fasting <5.5 mmol/L (<99 mg/dL), 1-hour <8.0 mmol/L (<144 mg/dL), and 2-hour postprandial <7.0 mmol/L (<126 mg/dL).

However, we found clinically important differences between the glycaemic treatment target groups in the prespecified analyses for some of the secondary outcomes. Firstly, use of tighter glycaemic targets reduced the risk of a serious health outcome for the infant after adjustment for gestational age at time of diagnosis of GDM, maternal BMI, ethnicity, and previous history of GDM. There were no stillbirths in the tighter target group compared to 3 in the less tight target group and no neonatal deaths in either glycaemic group. This suggests that the risk of serious infant outcome, including perinatal mortality, may be modifiable when tighter targets for glycaemic control are used. Clearly, this important finding needs to be assessed in other settings. Secondly, and unexpectedly, women with GDM randomised to tighter glycaemic targets had a higher incidence of a serious health outcome. The main contributor to this composite outcome was major postpartum haemorrhage, although none of the individual components of the composite outcome were statistically different between the 2 target glycaemic groups. GDM is associated with risk factors for postpartum haemorrhage, including macrosomia and operative birth, but these did not differ between the target groups. We are not aware of a direct association between GDM and major postpartum haemorrhage, and so the higher incidence of serious health outcomes we observed for these women remains unexplained. It will be important to assess this in other settings. Thirdly, women using the tighter treatment targets had greater use of pharmacological therapies, both oral metformin and insulin that would lead to an increase in healthcare costs. Previous randomised controlled trials of tighter glycaemic control, in type 1 [28] and type 2 [29] diabetes, and preterm babies [30] have shown an increased incidence of hypoglycaemia with tighter glycaemic control. Although the risk of maternal hypoglycaemia is increased when insulin is used compared with an oral hypoglycaemic agent, in our trial the incidence of maternal hypoglycaemia was low, even in women using the tighter glycaemic targets, and did not differ between glycaemic target groups. The length of stay in intensive care was shorter for infants whose mother had been in the tighter glycaemic target group, which would be associated with maternal emotional benefit, increased early mother–infant bonding and beneficial cost consequences. For the remaining secondary outcomes, we found no differences between use of the tighter and less tight targets; for the infant, these included hypoglycaemia, hyperbilirubinaemia and need for respiratory support and for the woman included preeclampsia, need for induction of labour and cesarean birth.

The major strengths of the TARGET Trial are that it is the largest randomised comparison reported to date comparing the short-term effects of 2 contemporary different glycaemic targets used worldwide for women with GDM, the trial includes a diverse population and provides data on key maternal and infant outcomes. The stepped-wedge randomised trial design was chosen to be able to assess the impact on health outcomes of the implementation of tighter glycaemic targets [16]. Rather than assessing different targets at the same time for individual women with GDM at the same facility, the study design enabled all hospitals to adopt the updated, recommended tighter targets over the course of the trial.

The main limitation of our trial is the generalisability of the findings to other populations as we assessed glycaemic targets in use within New Zealand with new, lower targets recommended in Australia and New Zealand [12,16]. Glycaemic targets vary worldwide due to the lack of high-quality evidence on which to base recommendations, so further randomised trials are warranted in countries using different glycaemic targets. A limitation was the imbalance for BMI, ethnicity, and history of GDM at baseline that occurred by chance that required further statistical adjustment as recommended [25], although the direction of effect and the overall findings remained unchanged.

Health practitioners currently using less tight glycaemic targets in their practice will find reassurance from our findings of similar neonatal outcomes, lower risk of serious maternal outcomes, and less need for pharmacological treatments for maternal blood glucose control. However, in view of our finding of an increased risk of a serious health outcome for infants when using less tight glycaemic targets, they will look for confirmation of these findings from further randomised trials. Health practitioners currently using tighter glycaemic targets in their practice, while finding some reassurance from our findings of fewer serious infant outcomes, will be concerned about the higher rate of serious maternal outcomes and increased need for pharmacological treatments for maternal blood glucose control that will require confirmation from further randomised trials.

Although our study was conducted in hospitals in a high-income country with a well-coordinated, publicly funded healthcare system, the results will be of relevance in other healthcare settings. Nevertheless, given the lack of high-quality trials to date for an increasingly common problem for pregnant women, [2] there is a need to confirm our findings by other randomised trials and in different healthcare settings. Previous data have been sparse, with our pretrial Cochrane systematic review [10] identifying only 1 abstract for a trial that reported data for 171 women and showed no differences between tighter or less tight targets for birth weight, macrosomia, gestational age at birth, or cesarean section, but an increase in use of insulin therapy with tighter targets [11]. We are aware of 2 other randomised trials comparing different glycaemic targets in women with GDM that have reported since our trial began; 1 trial [31] from Russia, reported only as an abstract to date, recruited 616 women with GDM, and 1 feasibility trial [32] from the USA that recruited 60 women with GDM. Neither trial found differences in perinatal outcomes between the different glycaemic targets groups, although both also showed an increase in the use of pharmacological therapies with the use of tighter targets.

In the TARGET Trial, the use of both tighter and less tight targets for glycaemic control in women with GDM resulted in a similar incidence of the infant being born large for gestational age and of other key neonatal and maternal outcomes including hypoglycaemia, hyperbilirubinaemia, and need for respiratory support, preeclampsia, need for induction of labour, and cesarean birth. However, tighter glycaemic targets compared to less tight targets reduced the risk of serious morbidity for the infant and shortened their length of stay in neonatal intensive care, but for the woman, there was an increase in the risk of a serious health outcome and an increased use of pharmacological therapy to treat their hyperglycaemia. These findings have direct relevance for clinical practice and can be used to aid decisions on the choice of treatment targets to use when discussing glycaemic control with women with GDM.

## Supporting information

S1 File TARGET Trial Protocol. (PDF)

S1 Table Consort Checklist. (DOCX)

S2 Table Sensitivity analysis with multiple random effects for infant outcomes. (DOCX)

S3 Table Sensitivity analysis with multiple random effects for maternal outcomes. (DOCX)

S4 Table TARGET Study Group. (DOCX)

## Abbreviations

adjRR: adjusted relative risk
CI: confidence interval
FPG: fasting plasma glucose
GDM: gestational diabetes mellitus
NNTB: number needed to treat to benefit
OGTT: oral glucose tolerance test
RR: relative risk
