# Supplementary material for: Tighter or less tight glycaemic targets for women with gestational diabetes mellitus for reducing maternal and perinatal morbidity: A stepped-wedge, cluster-randomised trial
Source: PLoS Med. 2022 Sep 8;19(9):e1004087. doi: 10.1371/journal.pmed.1004087 (PMC9455881; doi:10.1371/journal.pmed.1004087)
Supplement: S1 File — (PDF) [file pmed.1004087.s001.pdf]

Crowther et al. BMC Pregnancy and Childbirth (2018) 18:425
https://doi.org/10.1186/s12884-018-2060-2

BMC Pregnancy and Childbirth

STUDY PROTOCOL Open Access

Tight or less tight glycaemic targets for women with gestational diabetes mellitus for reducing maternal and perinatal morbidity? (TARGET): study protocol for a stepped wedge randomised trial

Caroline A. Crowther[1*], Jane M. Alsweiler[2,3], Ruth Hughes[4], Julie Brown[1] and for the Target Study Group

Abstract

Background: Gestational diabetes mellitus (GDM) is strongly associated with significant adverse maternal and perinatal health outcomes that have lifelong consequences. Treatment for women with GDM aims to normalise maternal blood glucose concentrations to reduce these adverse health risks. Target recommendations for glycaemic control in women with GDM vary amongst international organisations. All their recommendations rely on consensus, as there have been no published randomised trials that compare different intensities of glucose control in women with GDM. The TARGET Trial aims to determine whether tighter targets for glycaemic control in women with GDM compared with less tight targets, reduce maternal and perinatal morbidity without adverse health consequences.

Methods/design: Using a stepped wedge, cluster randomised trial the 10 participating hospitals will be randomised to the timing of the change from the less tight to the tighter glycaemic target period. During the less tight target period, all health professionals at the hospital will aim to use the less tight glycaemic targets for treatment of women with GDM (fasting plasma glucose < 5.5 mmol/L; 1 h postprandial < 8.0 mmol/L; 2 h postprandial < 7.0 mmol/L). During the tighter target period all health professionals at the hospital will aim to use the tighter glycaemic targets for treatment of women with GDM (fasting plasma glucose ≤5.0 mmol/L, 1 h postprandial ≤7.4 mmol/L; 2 h postprandial ≤6.7 mmol/L). The primary study outcome is large for gestational age infant (birth weight > 90th centile). A sample size of 1080 participants will detect a treatment difference of 6% in the proportion of large for gestational age babies from 13% with less tight glycaemic targets to 7% with tighter targets, assuming an intra-cluster correlation coefficient of 0.05.

Discussion: The TARGET Trial will provide high-level evidence of direct relevance for clinical practice. If tighter treatment targets for women with GDM clearly result in significantly fewer large for gestational age infants and less adverse maternal and perinatal outcomes then they should be recommended for women with GDM. This would be of great importance to these women, their children, health services and communities.

Trial registration: Australian New Zealand Clinical Trials Registry - ACTRN 12615000282583.

Keywords: Gestational diabetes mellitus, Maternal glycaemic targets, Large for gestational age, Stepped wedged cluster randomised trial

* Correspondence: c.crowther@auckland.ac.nz
[1]Liggins Institute, The University of Auckland, Building 503, Level 2, 85 Park Road, Private Bag 92019, Auckland 1142, New Zealand
Full list of author information is available at the end of the article

© The Author(s). 2018 **Open Access** This article is distributed under the terms of the Creative Commons Attribution 4.0 International License (http://creativecommons.org/licenses/by/4.0/), which permits unrestricted use, distribution, and reproduction in any medium, provided you give appropriate credit to the original author(s) and the source, provide a link to the Creative Commons license, and indicate if changes were made. The Creative Commons Public Domain Dedication waiver (http://creativecommons.org/publicdomain/zero/1.0/) applies to the data made available in this article, unless otherwise stated.

Background

Gestational diabetes mellitus (GDM) is defined by the World Health Organization as “carbohydrate intolerance resulting in hyperglycaemia with onset or first recognition during pregnancy” [1]. Rates for GDM are rising worldwide with significant variation in rates, reported as being between 5.2 to 25.5% depending on the diagnostic criteria used and the population [2–4]. With rates rising, GDM is a significant and increasing health problem in New Zealand, now affecting one in every 11 pregnant women or over 5,500 pregnant women every year [5, 6].

Risks during pregnancy for women with GDM include pre-eclampsia, induction of labour [7], increased likelihood of caesarean section and perineal trauma [8]. From a public health viewpoint the largest burden is that over half of the women diagnosed with GDM will develop type 2 diabetes within 10 years [9].

Relationship of maternal hyperglycaemia with severity of maternal and infant health risks

There is a continuous relationship between maternal glycaemia and the risk of neonatal macrosomia and hyperinsulinaemia [10]. Long-term effects on offspring adiposity and insulin sensitivity appear to be related to the degree of maternal glucose intolerance [11, 12]. The Hyperglycaemia and Adverse Pregnancy Outcomes (HAPO) cohort study [10] confirmed a strong, continuous association between higher concentrations of maternal glucose and increased birth weight and high cord-blood serum C-peptide levels, a marker for fetal hyperinsulinaemia.

Treatment of GDM improves maternal and infant health

Providing women with GDM with individualised dietary and lifestyle advice, blood glucose monitoring and pharmacological treatment when required, reduces the risk of serious perinatal outcomes, the likelihood of a larger for gestational age infant and improves the woman’s health related quality of life [7, 13]. Treatment for women with GDM aims to normalise maternal fasting and postprandial glucose concentrations, reducing fetal macrosomia related to fetal hyperglycaemia and fetal hyperinsulinaemia, and the associated neonatal complications.

How tight should glycaemic targets be for women with GDM?

Normal blood glucose values in the second half of pregnancy in non-obese, non-diabetic pregnant women have been determined using continuous glucose monitoring. The fasting plasma glucose was 4.2 mmol/L ± 0.7; 1 h postprandial plasma glucose 5.8 mmol/L ± 0.7; and 2 h postprandial plasma glucose 5.4 mmol/L ± 0.6 [14]. These values reflect blood glucose concentrations in women with a ‘normal’ pregnancy. These are all lower than the currently recommended glycaemic targets used for the treatment of women with GDM. The maternal glucose concentrations in non-diabetic pregnant women are considerably lower than the glycaemic targets used for treating women with GDM so the current recommendations for glycaemic targets for the treatment of women with GDM has recenlty been challenged [15].

Systematic review of glycaemic treatment targets for women with GDM

The evidence for glucose treatment targets in pregnant women with diabetes has been summarised in a systematic review that included 34 observational studies involving 9433 women [16]. Twenty-six of the studies included women with GDM, but only in the third trimester of pregnancy. The review authors highlighted that there have been no published randomised controlled trials comparing any two glycaemic thresholds that report on benefits and harms for the mother and infant [16]. The evidence able to be included within the systematic review was therefore only observational.

Overall the quality of the evidence included was judged to be low, with the literature limited in quality and heterogeneity found to be high amongst the studies. The studies had moderate to high risk of bias due to inconsistent reporting of outcomes and a lack of adjustment for important variables such as maternal body mass index [16].

The results of this systematic review showed that for women with GDM a fasting glucose treatment target of < 5.0 mmol/L was associated with a significant reduction in macrosomia ($p < 0.01$), large for gestational age infants ($p = 0.01$), neonatal hypoglycaemia (p = 0.01) and neonatal jaundice (p = 0.01) [16]. There was a significant reduction in pre-eclampsia during the third trimester of pregnancy (p = 0.01) [16].

The systematic review authors concluded that it remains unclear whether glycaemic treatment targets above or below a fasting glucose threshold of < 5.0 mmol/L offer a better balance of benefits and risks [16]. They considered that there was insufficient evidence on postprandial measures to assess different cut-off points and health outcomes.

What treatment targets for GDM are recommended for clinical practice?

Target recommendations from international professional organisations for maternal glycaemic control on GDM differ widely. All rely on consensus because there is a lack of high quality evidence [3, 17–20]. Treatment target recommendations for fasting glucose concentrations range from 3.5 to 5.9 mmol/L, for 1 h postprandial from 6.0 to < 8.0 mmol/L and 2 h postprandial from 5.6 to <

7.0 mmol/L. The evidence on which these recommendations have been made is generally unclear and does not compare different blood glucose thresholds at which to initiate treatment.

In New Zealand, the recommended targets for glucose for treatment of women with GDM have been a fasting plasma glucose < 5.5 mmol/L; 1 h postprandial < 8.0 mmol/L; and 2 h postprandial < 7.0 mmol/L [21]. With increasing concerns that these recommended glycaemic targets were not tight enough to normalise fetal growth and so minimise perinatal and later complications [11, 16, 17, 22, 23], the Ministry of Health Clinical Practice Guidelines "Screening, diagnosis and management of gestational diabetes in New Zealand," have recommended tighter targets [6]. There have been many calls for randomised trials to be conducted comparing different intensities of treatment targets [6, 15, 16, 20, 22, 23].

Aims and objectives of the TARGET trial
The TARGET Trial will assess whether tighter targets for glycaemic control in women with GDM, as now proposed by the New Zealand Ministry of Health [6], compared with less tight targets [21], reduce maternal and perinatal morbidity without adverse health consequences.

Hypotheses
The primary hypothesis of the trial is that tighter treatment targets for glycaemic control in women with GDM compared with less tight targets will reduce the risk of the infant being born large for gestational age.

The secondary hypotheses are that tighter treatment targets for glycaemic control in women with GDM compared with less tight targets will:

1. reduce the risk of serious morbidity for the infant (composite outcome measure of death, shoulder dystocia, birth trauma);
2. reduce other infant morbidity including hypoglycaemia, hyperbilirubinaemia and need for respiratory support;
3. reduce morbidity for the woman including preeclampsia, need for induction of labour and caesarean section;
4. increase the use of pharmacological treatment and hospital services.

Methods/design
Ethics statement
Human ethics approval was granted by the Northern A Health and Disability Ethics Committee in New Zealand (14/NTA/163/AMO1) that included a waiver of consent for eligible women being treated for gestational diabetes at the participating hospitals.

Study design
A multicentre, stepped wedge, cluster, randomised trial [24–26], protocol date 2014 version 1. This study design has been used mainly to evaluate interventions during routine implementation, particularly where there is a strong likelihood of benefit rather than harm [27]. Individual hospitals will be randomised rather than individual women allowing assessment of the benefits and harms of the less tight and tighter treatment targets. All hospitals will sequentially implement the tighter glycaemic treatment targets at randomly assigned time points. These time points are the "step" of the stepped-wedge design.

Study population and sites
Women with GDM, diagnosed by oral glucose tolerance test in mid-pregnancy, receiving pregnancy care at one of the 10 participating hospitals in New Zealand will be eligible. Women with a known major fetal anomaly will not be eligible. Eligible women will be enrolled into the study by the research assistant at each hospital over six time periods of four months each.

Randomisation
Hospitals will be randomised, in clusters of two, to the timing of the change from use of the less tight glycaemic target period to use of the tighter glycaemic target period. The sequence of allocation of the hospitals to the sequential implementation of the tighter glycaemic target period will be prepared by the trial statistician using a computer generated random number table. Participating sites will be blind to their randomised time point until training for the implementation of the tighter glycaemic targets begins at their site, not more than two weeks prior to the change.

For the less tight Glycaemic target period
All health professionals at the hospital will aim to use the less tight targets for glycaemic control in women with GDM (fasting plasma glucose < 5.5 mmol/L; 1 h postprandial < 8.0 mmol/L; 2 h postprandial < 7.0 mmol/L) [21].

For the tighter Glycaemic target period
All heatlh professionals at the hospital will aim to use the tighter targets for glycaemic control in women with GDM (fasting plasma glucose ≤5.0 mmol/L, 1 h postprandial ≤7.4 mmol/L; 2 h postprandial ≤6.7 mmol/L) [6, 20].

Procedures at the sites
Prior to the study commencing, a visit will be made to each of the sites by the lead investigator and the Study Co-ordinator. They will meet with the participating hospital's clinical leaders for the study to plan the set-up of the project at their site, and to provide the TARGET

Trial Implementation Action Pack. This pack includes the trial protocol, a powerpoint presentation about the study for use in local educational meetings, stickers of the less tight targets to be used in the participant's blood glucose monitoring booklets, lanyard cards for relevant hospital staff and posters to display in clinical areas giving the less tight targets for glycaemic control in use at the hospital. At the start of the Less Tight Glycaemic Target Period all health professionals at the participating hospitals caring for women with GDM will be sent a reminder of the less tight targets to use for glycaemic control in women with GDM.

Not more than two weeks prior to a site being randomised to the Tighter Glycaemic Target Period a visit will be made by the lead investigator and the Study Co-ordinator to plan the procedures for the change to use of the tighter targets with the local clinical collaborators and the hospital staff. Updated materials will be provided for the site's TARGET Trial Implementation Action Pack that include an updated powerpoint of the changed targets, stickers of the tighter targets to be used for the participant's blood glucose monitoring booklets, lanyard cards for relevant staff and posters to display in clinical areas giving the tighter glycaemic targets to be used. At the start of the Tighter Glycaemic Targets Period all health professionals at the participating hospitals caring for women with GDM will be sent a reminder of the tighter glycaemic targets to be used for glycaemic control in women with GDM.

For both Glycaemic target periods

Women with GDM attending the participating hospitals will receive standard management for GDM by their lead maternity carer and the local Diabetes Pregnancy Service, with appropriate dietary and lifestyle advice, blood glucose monitoring and further pharmacological treatment as needed. Care of the baby after birth will be according to the hospital protocol for blood glucose monitoring.

Data collection

At each of the participating sites a research assistant will collect the study outcome data from the case records of the women enrolled and their infants up to the time of primary hospital discharge after the birth. Data will be submitted to the study's data management centre at The Liggins Institute, University of Auckland and stored in a password protected database (target@auckland.ac.nz).

Primary study endpoint

The primary study endpoint is the incidence of large for gestational age - defined as a birth weight > 90th centile using growth charts adjusted for gestational age and infant sex [28].

Secondary study endpoints

For the woman

Secondary study endpoints for the women include a composite of serious maternal health outcomes; pre-eclampsia; induction of labour; caesarean section; use of pharmacological treatment for GDM; maternal hypoglycaemia; need for admission and length of any antenatal stay; length of postnatal stay; and breast feeding at hospital discharge.

For the infant

Secondary study endpoints for the infant include a composite of serious health outcomes - defined as perinatal death or birth trauma (nerve palsy, bone fracture), or shoulder dystocia; gestational age at birth; birth weight; macrosomia - defined as birth weight $\geq$ 4 kg; small for gestational age - defined as a birth weight < 10th centile using growth charts adjusted for gestational age and infant sex [28]; length at birth; head circumference at birth; use of respiratory support; hypoglycaemia; hyperbilirubinaemia; neonatal intensive care unit admission and length of stay; and length of postnatal stay.

Sample size

A total sample size of 1080 participants from 10 hospitals over 6 study periods of four months will provide 90% power at 5% level of significance (two-sided) to detect a treatment difference of 6% in the proportion of large for gestational age babies, from 13% [7] using the less tight glycaemic treatment targets to 7% [13] using the tighter glycaemic treatment targets, assuming an intra-cluster correlation coefficient of 0.05 [24].

Monitoring

An independent Data Monitoring Committee (DMC) with terms of reference will be established. The DMC will monitor the study processes and review any serious adverse events reported by the study sites. No interim analyses are planned.

Analysis and reporting of results

Data will be analysed by a statistician independent of the clinical investigators who will prepare the statistical analysis plan. Baseline characteristics of all women will be summarised descriptively by the two treatment periods, less tight and tighter glycaemic target periods, to assess comparability of the study groups. Data points in the control section wedge – Less Tight Target Period - will be compared with data points in the intervention section - Tighter Target Period - to assess the effectiveness of the intervention [29]. Treatment evaluations will follow the intention-to-treat principle. Statistical tests will be two-sided and maintained at 5% level of significance. Generalized linear mixed models will be used to evaluate the

main treatment effect, with a random effect for hospital group and fixed effects for the intervention implementation and the study time period. Both unadjusted and adjusted analyses will be carried out. Secondary exploratory analyses will consider baseline covariates that show evidence of imbalance between study groups and are related to the outcome of interest. The risk estimates and 95% confidence intervals will be reported using log binomial regression for binary outcomes. Continuous outcomes will be analysed using linear regression. All model assumptions, including normality, will be assessed.

Discussion

Gestational diabetes is a major and increasing health problem globally. Maternal risks include preeclampsia, induction of labour, and within 10 years, over half of the women will have developed type 2 diabetes. Infants born to mothers with GDM are more likely to be large for gestational age, which is highly correlated with the degree of maternal hyperglycaemia and strongly associated with birth injuries and caesarean birth [7, 13]. Large for gestational age offspring are at high risk of long-term adverse health that includes obesity, diabetes, and the metabolic syndrome [9]. Effective interventions that normalise maternal glycaemia and optimise fetal growth and so reduce the risk of being born large for gestational age have the potential for prevention of both short and long-term health problems.

Clinical practice recommendations on the glycaemic targets to use in the treatment of women with GDM vary worldwide and are based on consensus, as there have been no randomised trials published. The optimal glycaemic treatment targets to advise women with GDM to use are therefore unclear.

The TARGET Trial will provide high-level evidence of direct relevance for clinical practice. If tighter treatment targets for women with GDM results in significantly fewer large for gestational age infants and less adverse maternal and perinatal outcomes then they should be recommended for women with GDM and would be of great importance for these women, their children, health services and local communities.

Abbreviation
GDM: Gestational diabetes mellitus

Acknowledgements
None.

Funding
This study is funded through a 4-year project grant from the Health Research Council (HRC) in New Zealand (No. 14/449). The funding body had no role in the study design or writing of the manuscript.

Availability of data and materials
Data sharing is not applicable to this article as no datasets were generated or analysed during the current study.

Authors' contributions
CAC, JMA, RH, and JB are all members of the TARGET Trial Steering Group who will oversee the co-ordination of the trial and will be responsible for dissemination of the study results. The primary investigator of the TARGET Trial (CAC) wrote the study protocol and prepared the initial draft of this manuscript. CAC and JB conceived the study. All authors were involved in the development of the design of the study, the protocol development, have commented on all drafts of the protocol and have read and approved the final draft of this manuscript. The Target Study Group will consist of investigators and research staff at the study sites.

Ethics approval and consent to participate
Human ethics approval was granted by the Northern A Health and Disability Ethics Committee in New Zealand (14/NTA/163/AMO1) that included a waiver of consent for eligible women being treated for gestational diabetes at the participating hospitals.

Consent for publication
Not applicable.

Competing interests
The authors declare they have no competing interests.

Publisher's Note
Springer Nature remains neutral with regard to jurisdictional claims in published maps and institutional affiliations.

Author details
[1]Liggins Institute, The University of Auckland, Building 503, Level 2, 85 Park Road, Private Bag 92019, Auckland 1142, New Zealand. [2]Newborn Services, Auckland City Hospital, Auckland, New Zealand. [3]Department of Paediatrics: Child and Youth Health, University of Auckland, Auckland, New Zealand. [4]Department of Obstetrics and Gynaecology, Christchurch Women's Hospital, University of Otago, Christchurch, New Zealand.

Received: 25 September 2017 Accepted: 16 October 2018
Published online: 29 October 2018

References
1. Alberti K, Zimmet P. Definition, diagnosis and classification of diabetes mellitus. Part 1: diagnosis and classification of diabetes mellitus: World Health Organisation report. Diabet Med. 1998;15(7):539–53.
2. Cheung NW, Byth K. Population health significance of gestational diabetes. Diabetes Care. 2003;26(7):2005–9.
3. National Collaborating Centre for Women's and Children's Health (UK). Diabetes in Pregnancy: Management of Diabetes and its Complications from Preconception to the Postnatal Period. London: Royal College of Obstetricians and Gynaecologists; 2008.
4. Sacks DA, Hadden DR, Maresh M, Deerochanawong C, Dyer AR, Metzger BE, et al. For the HAPO study cooperative research group. Frequency of gestational diabetes mellitus at collaborating centers based on IADPSG consensus panel-recommended criteria: the hyperglycemia and adverse pregnancy outcome (HAPO) study. Diabetes Care. 2012;35:526–8.
5. Auckland District Health Board. National Women's Hospital. National Women's Annual Clinical Report. Auckland: National Women's Health, Auckland District Health Board; 2017. ISSN 1175-6667. (http://nationalwomenshealth.adhb.govt.nz
6. Ministry of Health. Guideline for the screening, diagnosis and treatment of women with gestational diabetes in New Zealand - Ministry of Health; 2014.
7. Crowther CA, Hiller JE, Moss JR, McPhee AJ, Jeffries WS, Robinson JS. The Australian carbohydrate intolerance study in pregnant women (ACHOIS) trial group. Effect of treatment of gestational diabetes mellitus on pregnancy outcomes. N Engl J Med. 2005;352(24):2477–86.
8. Dodd JM, Crowther CA, Antoniou G, Baghurst P, Robinson JS. Screening for gestational diabetes: the effect of varying blood glucose definitions in the prediction of adverse maternal and infant health outcomes. Aust N Z J Obstet Gynaecol. 2007;47(4):307–12.
9. Boney CM, Verma A, Tucker R, Vohr BR. Metabolic syndrome in childhood: association with birth weight, maternal obesity, and gestational diabetes mellitus. Pediatrics. 2005;115(3):e290–6.
10. The HAPO Study Cooperative Research Group. Hyperglycemia and adverse pregnancy outcomes. N Engl J Med. 2008;358(19):1991–2002.

11. Catalano PM, Thomas A, Huston-Presley L, Amini SB. Increased fetal adiposity: a very sensitive marker of abnormal in utero development. Am J Obstet Gynecol. 2003;189(6):1698–704.
12. Franks PW, Looker HC, Kobes S, Touger L, Tataranni PA, Hanson RL, et al. Gestational glucose tolerance and risk of type 2 diabetes in young Pima Indian offspring. Diabetes. 2006;55(2):460–5.
13. Landon MB, Spong CY, Thom E, Carpenter MW, Ramin SM, Casey B, et al. For the Eunice Kennedy Shriver National Institute of child and human development maternal-fetal medicine units. A multicenter, randomized trial of treatment for mild gestational diabetes. N Engl J Med. 2009;361(14): 1339–48.
14. Yogev Y, Ben-Haroush A, Chen R, Rosenn B, Hod M, Langer O. Diurnal glycemic profile in obese and normal weight non-diabetic pregnant women. Am J Obstet Gynecol. 2004;191:949–53.
15. Hernandez TL, Friedman JE, Van Pelt RE, Barbour LA. Patterns of Glycemia in Normal pregnancy: should the current therapeutic targets be challenged? Diabetes Care. 2011;34(7):1660–8.
16. Prutsky GJ, Domecq JP, Wang Z, Carranza Leon BG, Elraiyah T, Nabhan M, et al. Glucose targets in pregnant women with diabetes: a systematic review and meta-analysis. J Clin Endocrinol Metab. 2013;98(11):4319–24.
17. Metzger BE, Buchanan TA, Coustan DR, de Leiva A, Dunger DB, Hadden DR, et al. Summary and recommendations of the fifth international workshop-conference on gestational diabetes mellitus. Diabetes Care. 2007;30(Suppl 2):S251–60.
18. Network SIG. Management of diabetes. A national clinical guideline. Edinburgh, Scotland: Scottish Intercollegiate Guidelines Network; 2010.
19. American Diabetes Association. Standards of medical Care in Diabetes - 2013. Diabetes Care. 2013;36(Suppl 1):S11–66.
20. Nankervis A, McIntyre HD, Moses R, Ross GP, Callaway L, Porter C, et al. For the Australasian Debetes in Pregnancy Society. Australasian Diabetes In Pregnancy Society (ADIPS) Consensus Guidelines for the Testing and Diagnosis of Gestational Diabetes Mellitus in Australia. 2013.
21. Hoffman L, Nolan C, Wilson JD, Oats JJ, Simmons D. Gestational diabetes mellitus--management guidelines. The Australasian diabetes in pregnancy society. Med J Aust. 1998;169(2):93–7.
22. Combs CA, Moses RG. Aiming at new targets to achieve Normoglycemia during pregnancy. Diabetes Care. 2011;34(10):2331–2.
23. IADPSG Consensus Panel. International Association of Diabetes and Pregnancy Study Groups Recommendations on the diagnosis and classification of hyperglycemia in pregnancy. Diabetes Care 2010;33(3): 676–82.
24. Hussey M, Hughes J. Design and analysis of stepped wedge cluster randomized trials. Contemp Clin Trials. 2007;28(2):182–91.
25. Mdege ND, Man M-S, Taylor CA, Torgerson DJ. Systematic review of stepped wedge cluster randomized trials shows that design is particularly used to evaluate interventions during routine implementation. J Clin Epidemiol. 2011;64(9):936–48.
26. Woertman W. Stepped wedge designs could reduce the required sample size in cluster randomized trials. J Clini Epidemiol. 2013;66(7):752–8.
27. Hughes J, Goldenberg RL, Wilfert CM, Valentine M, Mwinga KG, Guay LA, et al. Design of the HIV prevention trials network (HPTN) protocol 054: a cluster randomised crossover trial to evaluate combined access to Nevirapine in developing countries. UW Biostatistics Working Paper Series. 2003.
28. Fenton TR, Kim JH. A systematic review and meta-analysis to revise the Fenton growth charts for preterm infants. BMC Pediatr. 2013;13:59.
29. Brown CA, Lilford RJ. The stepped wedge design: a systematic review. BMC Med Res Methodol. 2006;6:54.

Ready to submit your research? Choose BMC and benefit from:

- fast, convenient online submission
- thorough peer review by experienced researchers in your field
- rapid publication on acceptance
- support for research data, including large and complex data types
- gold Open Access which fosters wider collaboration and increased citations
- maximum visibility for your research: over 100M website views per year

At BMC, research is always in progress.

Learn more biomedcentral.com/submissions

BMC
