# Supplementary material for: Tighter or less tight glycaemic targets for women with gestational diabetes mellitus for reducing maternal and perinatal morbidity: A stepped-wedge, cluster-randomised trial
Source: PLoS Med. 2022 Sep 8;19(9):e1004087. doi: 10.1371/journal.pmed.1004087 (PMC9455881; doi:10.1371/journal.pmed.1004087)
Supplement: S2 Table — (DOCX) [file pmed.1004087.s003.docx]

**S2 Table. Sensitivity analysis with multiple random effects for infant outcomes**

| **Outcomes** | **Tighter targets (n=599)** | **Current targets (n=502)** | | **Treatment effects^+^ (95% CI)** | **P value^+^** | **Adjusted^+^ * Treatment effects (95% CI)** | **Adjusted^+^* P value** | | |
| --- | --- | --- | --- | --- | --- | --- | --- | --- | --- |
| Large for gestational age | 88/599 (14.7%) | 76/502 (15.1%) | | 0.97 (0.66- 1.41) | 0.867 | 0.96 (0.66- 1.40) | 0.849 | | |
| Birth weight (g)^1^ | 3367.95 (537.94) | 3359.29 (557.68) | | 25.39 (-62.51- 113.29) | 0.602 | 20.52 (-59.63- 100.68) | 0.642 | | |
| Birth weight (z score)^1^ | 0.32 (1.02) | 0.22 (1.02) | | 0.14 (-0.06- 0.33) | 0.245 | 0.12 (-0.06- 0.30) | 0.247 | | |
| Small for gestational age | 29/599 (4.8%) | 31/502 (6.2%) | | 0.63 (0.33- 1.20) | 0.235 | 0.64 (0.33- 1.21) | 0.241 | | |
| Macrosomia | 61/599 (10.2%) | 54/502 (10.8%) | | 1.11 (0.72- 1.73) | 0.657 | 1.10 (0.71- 1.72) | 0.685 | | |
| Length at birth- large for gestational age | 120/556 (21.6%) | 100/438 (22.8%) | | 0.98 (0.71- 1.36) | 0.913 | 0.98 (0.71- 1.36) | 0.921 | | |
| Length at birth (cm)^1^ | 50.81 (2.65) | 50.91 (2.65) | | 0.13 (-0.28- 0.53) | 0.566 | 0.12 (-0.29- 0.52) | 0.603 | | |
| Birth length (z score)^1^ | 0.57 (1.01) | 0.56 (1.02) | | 0.12 (-0.04- 0.27) | 0.206 | 0.12 (-0.04- 0.27) | 0.209 | | |
| Head circumference at birth- large for gestational age | 112/560 (20.0%) | 84/456 (18.4%) | | 1.00 (0.71- 1.42) | 0.983 | 1.00 (0.71- 1.42) | 0.997 | | |
| Birth head circumference (cm)^1^ | 34.58 (1.57) | 34.50 (1.72) | | 0.03 (-0.22- 0.28) | 0.834 | 0.02 (-0.23- 0.26) | 0.892 | | |
| Birth head circumference (z score)^1^ | 0.40 (1.05) | 0.28 (1.10) | | 0.07 (-0.10- 0.25) | 0.454 | 0.07 (-0.10- 0.24) | 0.457 | | |
| Gestational age at birth (weeks)^1^ | 38.44 (1.30) | 38.61 (1.42) | -0.12 (-0.32- 0.07) | | 0.286 | -0.13 (-0.32- 0.07) | | 0.269 |  |
| Composite of serious health outcome | 8/599 (1.3%) | 13/505 (2.6%) | 0.34 (0.10- 1.10) | | 0.146 | 0.33 (0.10- 1.08) | | 0.142 |  |
| Stillbirth | 0/599 (0.0%) | 3/505 (0.6%) | N/A | | 0.095^ | N/A | | N/A |  |
| Neonatal death | 0/599 (0.0%) | 0/502 (0.0%) | N/A | | N/A | N/A | | N/A |  |
| Birth trauma | 0/599 (0.0%) | 0/502 (0.0%) | N/A | | N/A | N/A | | N/A |  |
| Shoulder dystocia | 8/599 (1.3%) | 10/502 (2.0%) | 0.45 (0.13- 1.55) | | 0.276 | 0.44 (0.13- 1.52) | | 0.267 |  |
| Use of respiratory support^2^ | 43/599 (7.2%) | 29/502 (5.8%) | 1.11 (0.62- 1.98) | | 0.716 | 1.13 (0.63- 2.01) | | 0.684 |  |
| Hypoglycaemia^2^ | 169/599 (28.2%) | 127/502 (25.3%) | 0.93 (0.69- 1.23) | | 0.594 | 0.92 (0.69- 1.23) | | 0.593 |  |
| Hyperbilirubinemia | 27/599 (4.5%) | 25/502 (5.0%) | 0.84 (0.42- 1.65) | | 0.633 | 0.83 (0.42- 1.64) | | 0.621 |  |
| Admission to neonatal intensive care unit^2^ | 22/599 (3.7%) | 20/502 (4.0%) | 0.52 (0.25- 1.07) | | 0.076 | 0.51 (0.25- 1.06) | | 0.073 |  |
| Length of stay of infants admitted to neonatal intensive care unit^2,3^ | 3.49 (4.55) | 5.10 (5.72) | 0.46 (0.23-0.93) | | 0.037 | 0.49 (0.26- 0.95) | | 0.041 |  |
| Length of postnatal stay (days)^2^ | 4.11 (4.75) | 4.18 (6.68) | 0.95 (0.84-1.08) | | 0.407 | 0.95 (0.84-1.08) | | 0.468 |  |

Data presented as number (%), and the treatment effects are relative risk (95% CI) estimated from the generalised linear mixed-effects model, with random effects for hospital groups, the time-by-hospital interaction and the interaction-by-hospital interaction, and fixed effects for the intervention and time interval between the assigned targets initiated and a woman recruited, unless otherwise indicated.

**^+^**: adjusted for clustering effect of infants within mothers.

*: adjusted for gestational age at oral glucose tolerance test (weeks).

^1^: data presented as mean (SD), and the treatment effects are mean difference (95% CI).

^2^: The model with a random effect for hospital groups was used as the model with multiple random effects did not converge.

^3^: data presented as mean (SD), and the treatment effects are mean ratio (95% CI).

^: mid-P exact test.

N/A: denotes none or too few events for the analysis to be done.
