# Supplementary material for: Tighter or less tight glycaemic targets for women with gestational diabetes mellitus for reducing maternal and perinatal morbidity: A stepped-wedge, cluster-randomised trial
Source: PLoS Med. 2022 Sep 8;19(9):e1004087. doi: 10.1371/journal.pmed.1004087 (PMC9455881; doi:10.1371/journal.pmed.1004087)
Supplement: S3 Table — (DOCX) [file pmed.1004087.s004.docx]

**S3 Table. Sensitivity analysis with multiple random effects for maternal outcomes**

| **Outcomes** | **Tighter targets (n=595)** | **Current targets (n=501)** | **Treatment effects (95% CI)** | **p-value** | **Adjusted* treatment effects (95% CI)** | **Adjusted* p-value** |
| --- | --- | --- | --- | --- | --- | --- |
| Serious maternal health outcome | 35/595 (5.9%) | 15/501 (3.0%) | 2.30 (0.91- 5.83) | 0.079 | 2.29 (0.90- 5.83) | 0.080 |
| Preeclampsia^1^ | 31/595 (5.2%) | 18/501 (3.6%) | 1.56 (0.78- 3.12) | 0.212 | 1.57 (0.78- 3.16) | 0.205 |
| Induction of labor^1^ | 312/595 (52.4%) | 259/501 (51.7%) | 0.96 (0.78-1.17) | 0.681 | 0.96 (0.78-1.17) | 0.681 |
| Caesarean delivery | 226/595 (38.0%) | 174/501 (34.7%) | 1.01 (0.79- 1.29) | 0.919 | 1.01 (0.80- 1.29) | 0.917 |
| Use of pharmacological treatment^1^ | 404/595 (67.9%) | 293/501 (58.5%) | 1.22 (1.01- 1.47) | 0.035 | 1.20 (1.00- 1.44) | 0.047 |
| Metformin^1^ | 330/595 (55.5%) | 213/501 (42.5%) | 1.25 (1.01- 1.54) | 0.036 | 1.25 (1.02- 1.54) | 0.036 |
| Insulin^1^ | 204/595 (34.3%) | 162/501 (32.3%) | 1.46 (1.14- 1.86) | 0.003 | 1.46 (1.14- 1.86) | 0.003 |
| Metformin and insulin^1^ | 130/598 (21.7%) | 82/501 (16.3%) | 1.65 (1.20-2.26) | 0.002 | 1.64 (1.19-2.26) | 0.002 |
| Maternal hypoglycaemia^1^ | 5/595 (0.8%) | 6/501 (1.2%) | 1.24 (0.31- 5.01) | 0.761 | 1.23 (0.31- 4.93) | 0.768 |
| Need for antenatal hospitalisation^1^ | 117/595 (19.7%) | 96/501 (19.2%) | 1.09 (0.79- 1.51) | 0.605 | 1.09 (0.79- 1.52) | 0.601 |
| Length of antenatal hospital stay (days)^1, 2^ | 3.85 (4.06) | 3.80 (3.14) | 0.95 (0.73- 1.23) | 0.685 | 0.96 (0.74- 1.25) | 0.757 |
| Length of postnatal stay (days)^2^ | 2.72 (1.85) | 2.66 (1.78) | 0.97 (0.88- 1.07) | 0.629 | 0.98 (0.89- 1.07) | 0.636 |
| Breastfeeding at discharge^1^ | 561/595 (94.3%) | 479/498 (96.2%) | 0.99 (0.85- 1.15) | 0.890 | 0.99 (0.85- 1.15) | 0.888 |

Data presented as number (%), and the treatment effects are relative risk (95% CI) estimated from the generalised linear mixed-effects model, with random effects for hospital groups, the time-by-hospital interaction and the interaction-by-hospital interaction, and fixed effects for the intervention and time interval between the assigned targets initiated and a woman recruited, unless otherwise indicated.

*: adjusted for gestational age at oral glucose tolerance test (weeks).

^1^: The model with a random effect for hospital groups was used as the complex model with multiple random effects did not converge.

^2^: data presented as mean (SD), and the treatment effects are mean ratio (95% CI).
