# Supplementary material for: Tighter or less tight glycaemic targets for women with gestational diabetes mellitus for reducing maternal and perinatal morbidity: A stepped-wedge, cluster-randomised trial
Source: PLoS Med. 2022 Sep 8;19(9):e1004087. doi: 10.1371/journal.pmed.1004087 (PMC9455881; doi:10.1371/journal.pmed.1004087)
Supplement: S4 Table — (DOCX) [file pmed.1004087.s005.docx]

TARGET Study Group

| Caroline | Crowther |
| --- | --- |
| Debbie | Samuel |
| Olga | Ksionda |
| Katie | Edwards |
| Ruth | Martis |
| Ruth | Hughes |
| Julie | Brown |
| Jane | Alsweiler |
| Thach | Tran |
| Vincent | Ball |
| Jane | Yates |
| Sarah | Philipsen |
| Sasha | Zhang |
| Vicki | Flenady |
| Lesley | McCowan |
| Greg | Gamble |
| Christopher | McKinlay |
| Dianne | Leishman |
| Joanna | Gullam |
| Kay | Faulls |
| Cate | Wilson |
| Stephanie | Farrand |
| Louise | Gelling |
| Heather | Charteris |
| Kirsten | Crawford |
| Robert | Leikis |
| Chloe | Goodson |
| Gesina | Marae |
| Kara | Okesene-Gafa |
| Susan | Edwards |
| John | Griffiths |
| Helen | Tippler |
| Pamela | Hale |
| Pauline | Tout |
| Rose | O’Connor |
| Sylvia | Keller |
| Anne | Faulkner |
| Fiona | Hamilton |
| Lewese | Hicks |
| Nic | Crook |
| Belinda | Chapman |
| Laird | Madison |
| Sandra | Luxton |
| Suzanne | Berry |
| Chris | Thurnell |
| Kingsley | Nirmalaraj |
| Louise | Maltby |
| Maureen | Alleyne |
| Miranda | McDonald Brown |
| Anne | Dymond |
| Donna | Madden |
| Lee | Alcock |
| Meggan | Zsemlye |
| Michelle | Downie |
| Eve | de Goey |
| Nicole | McGrath |
